# Supplementary material for: The binding and mechanism of a positive allosteric modulator of Kv3 channels
Source: Nat Commun. 2024 Mar 21;15:2533. doi: 10.1038/s41467-024-46813-8 (PMC10957983; doi:10.1038/s41467-024-46813-8)
Supplement: Supplementary file 1 — Supplementary Information [file 41467_2024_46813_MOESM1_ESM.docx]

**Supplementary Information**

**The binding and mechanism of a positive allosteric modulator of Kv3 channels.**

**Authors**

Qiansheng Liang,^1,2^ Gamma Chi,^3^ Leonardo Cirqueira,^4^ Lianteng Zhi,^1,2^ Agostino Marasco,^5^ Nadia Pilati,^5^ Martin J. Gunthorpe,^6^ Giuseppe Alvaro,^5^ Charles H. Large,^6^ David B. Sauer,^3^ Werner Treptow,^4^ Manuel Covarrubias^1,2*^

**Affiliations**

^1^Department of Neuroscience, ^2^Jack and Vicki Farber Institute for Neuroscience and the Jefferson Synaptic Biology Center, Sidney Kimmel Medical College of Thomas Jefferson University, Philadelphia, PA 19107.

^3^Centre for Medicines Discovery, Nuffield Department of Medicine, University of Oxford, Roosevelt Drive, Oxford, OX3 7DQ, United Kingdom.

^4^Laboratorio de Biologia Teorica e Computacional, University of Brasilia, Brasilia, Brazil

^5^Autifony Srl, Istituto di Ricerca Pediatrica Citta’ della Speranza, Via Corso Stati Uniti, 4f. 35127 Padua, Italy.

^6^Autifony Therapeutics, Ltd, Stevenage Bioscience Catalyst, Gunnels Wood Road, Stevenage, SG1 2FX, United Kingdom.

These authors contributed equally: Qiansheng Liang and Gamma Chi

***** Corresponding Author. [Manuel.Covarrubias@jefferson.edu](mailto:Manuel.Covarrubias@jefferson.edu)

**Table of Contents**

Figure S1 …………………………………………………………………………………………………………... 4 AUT5 is a highly selective positive modulator of Kv3.1 and Kv3.2 channels.

Figure S2…………………………………………………………………………………………………………… 5

Statistical analysis of AUT5-induced changes in the gating parameters of Shaker-related Kv channels.

Figure S3 …………………………………………………………………………………………………………... 7

The chemical groups of AUT1 and AUT5 and the positive modulation of Kv3.1a and Kv3.2a by AUT1

Figure S4 …………………………………………………………………………………………………………... 8

Kv3.4 channels stably expressed in HEK293 cells exhibit low sensitivity to AUT5.

Figure S5 …………………………………………………………………………………………………………... 9

Kv3.1 and Kv3.4 co-expressed in *Xenopus* oocytes form heteromultimers with reduced sensitivity to AUT5.

Figure S6 …………………………………………………………………………………………………………. 11

Cryo-EM data processing of the Kv3.1/AUT1 and Kv3.1/AUT5 complexes.

Figure S7 …………………………………………………………………………………………………………. 12

Additional structural analysis of the Kv3.1/AUT1 and Kv3.1/AUT5 complexes.

Figure S8 …………………………………………………………………………………………………………. 14

Cryo-EM map and model of the Kv3.1/AUT5 complex selectivity filter and voltage sensor residues.

Figure S9 …………………………………………………………………………………………………………. 15

Sequence alignments of Kv channel amino acid sequences.

Figure S10 ………………………………………………………………………………………………………... 17

Amino acid sequence divergence between Kv3.1 and Kv3.4 with structural context.

Figure S11 ………………………………………………………………………………………………………... 18

Blind docking calculations of AUT5 binding to Kv3.1 and Kv3.2.

Figure S12 ………………………………………………………………………………………………………... 19

Distribution of binding energy values for all suggested docking sites.

Figure S13 ……………………………………………………………………………………………………….. 20

Mutational and functional analyses of AUT5 and AUT1 binding site determinants.

Figure S14 ………………………………………………………………………………………………………... 21

The effects of deleting the T1 domain and swapping the extracellular S1S2 linker on the basal gating properties and AUT5 sensitivity of Kv3.2.

Figure S15 ………………………………………………………………………………………………………... 22

The effect of various Kv3.2 turret mutations on voltage-dependent gating under basal conditions.

Figure S16 ………………………………………………………………………………………………………... 23

The effect of various Kv3.4 turret mutations on voltage-dependent gating under basal conditions.

Figure S17 ………………………………………………………………………………………………………... 24

The effects of AUT5 following elimination of fast Kv3.4 inactivation by activation of PKC.

Figure S18 ………………………………………………………………………………………………………... 25

Gating properties of Kv3.2 mutants before and after bath application of 2 μM AUT5.

Figure S19 ……………………………………………………………………………………………………….. 29

Gating properties of Kv3.4 mutants before and after application of 2 μM AUT5 and in the presence of PMA.

Figure S20 ……………………………………………………………………………………………………….. 31

Turret mutations disrupt key interactions with the VSD without altering the AUT5 binding site.

Figure S21 ……………………………………………………………………………………………………….. 33

Comparison of the cryo-EM structures of the Kv3.1/AUT5 and Kv3.1/Compound-4 complexes.

Table S1…………………………………………………………………………………………………………... 34

Cryo-EM data collection, processing parameters and model refinement statistics.

Table S2…………………………………………………………………………………………………………... 35

Definitions of Kv3.2 and Kv3.4 mutations.

Table S3…………………………………………………………………………………………………………... 36

Mutagenesis primers

Table S4…………………………………………………………………………………………………………... 40

Summary of gating parameters derived from Kv3.1a binding site mutants.

Table S5…………………………………………………………………………………………………………... 41

Reagents.

Table S6…………………………………………………………………………………………………………... 42

Best-fit parameters of the G_p_-V_c_ curves for Kv3.2 shown on Fig. 1b.

Supplemental Results and Discussion …………………………………………………………………………… 43

Supplemental Methods …………………………………………………………………………………………... 46

Supplemental References ………………………………………………………………………………………... 48

**

**

**Figure S1. AUT5 is a highly selective positive modulator of Kv3.1 and Kv3.2 channels.** (**a**)-(**c**) and (**e**)-(**h**) Representative families of whole-oocyte currents before (black) and after (red) bath application of 2 μM AUT5, and the corresponding G_p_-V_c_ curves (Methods). Scale bars represent 1 µA and 100 ms. SEM bars are generally smaller than the symbols. (**d**) The voltage pulse protocol used to evoke the currents. The first and last voltage step were adjusted depending on the voltage range over which the Kv channel undergoes voltage-dependent activation in the absence or presence of the compound. (**i**) Scatter plots of the AUT5-induced changes in V_0.5_, z and G_max_. Each symbol represents a measurement from an individual oocyte. Short vertical bars indicate the mean values. For each tested Kv channel, P values evaluate differences relative to Kv3.2 (Kruskal-Wallis).








**Figure S2. Statistical analysis of AUT5-induced changes in the gating parameters of Shaker-related Kv channels.** The V_0.5_, G_max_ and z were obtained from the best fits of the Boltzmann equation (Methods). Each pair of connected symbols (before, black and after, red AUT5) represents the result from individual oocytes expressing the indicated Kv channel. The two-sided paired Student t-test was used to evaluate the significance of the changes. P values are indicated above each plot. The sample size was indicted in Fig S1

**Figure S3. The chemical groups of AUT1 and AUT5 and the modulations of Kv3.1 and Kv3.2 by AUT1.** (**a**) Chemical groups of AUT1 and AUT5. OP = oxypyridin-2-yl; ID = imidazoline-2,4-dione; MMPh = 3-methoxy-4-methylphenyl; and SBC = spiro[2H-1-benzofuran-3,1'-cyclopropane]-4-yl. The OP and ID groups are identical in AUT1 and AUT5. (**b**) Top: Families of Kv3.1 currents expressed in *Xenopus* oocytes, before and after exposure to 30 μM AUT1 (left and center). The voltage protocol is shown on Fig. S1d and described in the corresponding legend. The corresponding aggregate analysis of the corresponding G_p_ – V_c_ curves is also shown (right), including the best-fit Boltzmann function (solid line). (**b**) Bottom: as described for Kv3.1 above. The AUT1-induced ΔV_0.5_ values for Kv3.1 and Kv3.2 were -13.1±0.8 mV and 15.9±1.2 mV, respectively. Other parameters are reported in Table S4. At six-times lower concentration (5 μM), AUT5 induces a ΔV_0.5_ = -15.0 ± 0.4 mV of Kv3.1 (Fig. 4, main text), which approximately matches the change induced by 30 μM AUT1.











**

**

**Figure S4. Kv3.4 channels stably expressed in HEK293 cells exhibit low sensitivity to AUT5**. (**a**) Families of whole-cell currents before (top, black) and after bath application of 2 μM AUT5 (bottom, red). Currents were evoked by the pulse protocol shown on Fig. S1d and described in the corresponding legend. (**b**) Overlay of G_p_ – V_c_ curves under the conditions indicated on the graph. Solid lines are the best fits of the 1^st^ order Boltzmann equation. (**c**) Scatter plots of the changes in the best fit parameters V_0.5_, G_max_ and z. Each pair of connected symbols (before and after 2 μM AUT5) represents a measurement from an individual cell, and the bar represents the mean. The two-sided paired Student t-test was used to evaluate the significance of the changes. P values are indicated above each plot. The small hyperpolarizing shift induced by AUT5 was not significant (ΔV_0.5_ = -6.7±3.3 mV; P=0.11, n=5). (**d**) – (**f**) Conditions like those described for panels (a) - (c), except that the recordings were conducted before and after bath application of 10 μM AUT5.





**Figure S5. Kv3.1 and Kv3.4 co-expressed in *Xenopus* oocytes can form heteromultimers with reduced sensitivity to AUT5.** (**a**) Representative families of whole-oocyte currents recorded before (black) and after (red) application to 2 μM AUT5 from oocytes expressing Kv3.1 (left), Kv3.1/Kv3.4 heteromultimers (center) and Kv3.4 (right). 1.66 ng mRNA was injected into each oocyte and Kv3.1/Kv3.4 heteromultimers were 1:1 mixture of mRNA. All current families were evoked by the voltage protocol shown on Fig. S1d and described in the corresponding legend. Scale bars represent 1 μA and 100 ms. (**b**) Normalized aggregate G_p_ – V_c_ curves from the groups indicated in (a). Note that the curve from oocytes co-expressing Kv3.1 and Kv3.4 exhibits a voltage dependence that is intermediate between that of Kv3.1 and Kv3.4, as it expected for the expression of heteromultimeric complexes. (**c**) Voltage dependence of the time constants of macroscopic inactivation. The uniformly slower time constants of the Kv3.1/Kv3.4 group is also consistent with the formation of heteromultimers composed of Kv3.1 and Kv3.4 subunits. (**d**) Aggregate G_p_ – V_c_ curves derived from families of currents like those shown in (a) before and after application to 2 μM AUT5. Whereas Kv3.1 exhibits the expected AUT5-induced hyperpolarizing shift and Kv3.4 exhibits no significant shift (main text Fig. 1 and Fig. S1), the Kv3.1/Kv3.4 heteromultimers exhibit a significantly reduced hyperpolarizing shift. Δ*V*_0.5_ = -12.0±0.7 mV (Kv3.1, *n* = 9), -6.1±0.8 mV (Kv3.1/Kv3.4, *n* = 12), and 1.2±0.8 mV (Kv3.4, *n* = 7). (**e**) Normalized aggregate G_s_ – V_c_ curves derived from sustained currents measured at the end of the trace before and after application to 2 μM AUT5. Because there is a greater contribution of Kv3.1 homomultimers to the sustained current, the AUT5-induced hyperpolarizing shifts of the Kv3.1 and Kv3.1/Kv3.4 groups are similar. Δ*V*_0.5_ = -12.7±0.7 mV (Kv3.1, *n* = 9) and -10.4±0.4 mV (Kv3.1/Kv3.4, *n* = 12). Solid lines in (b), (d) and (e) are the best fits of the 1^st^-order Boltzmann equation (Methods).

**
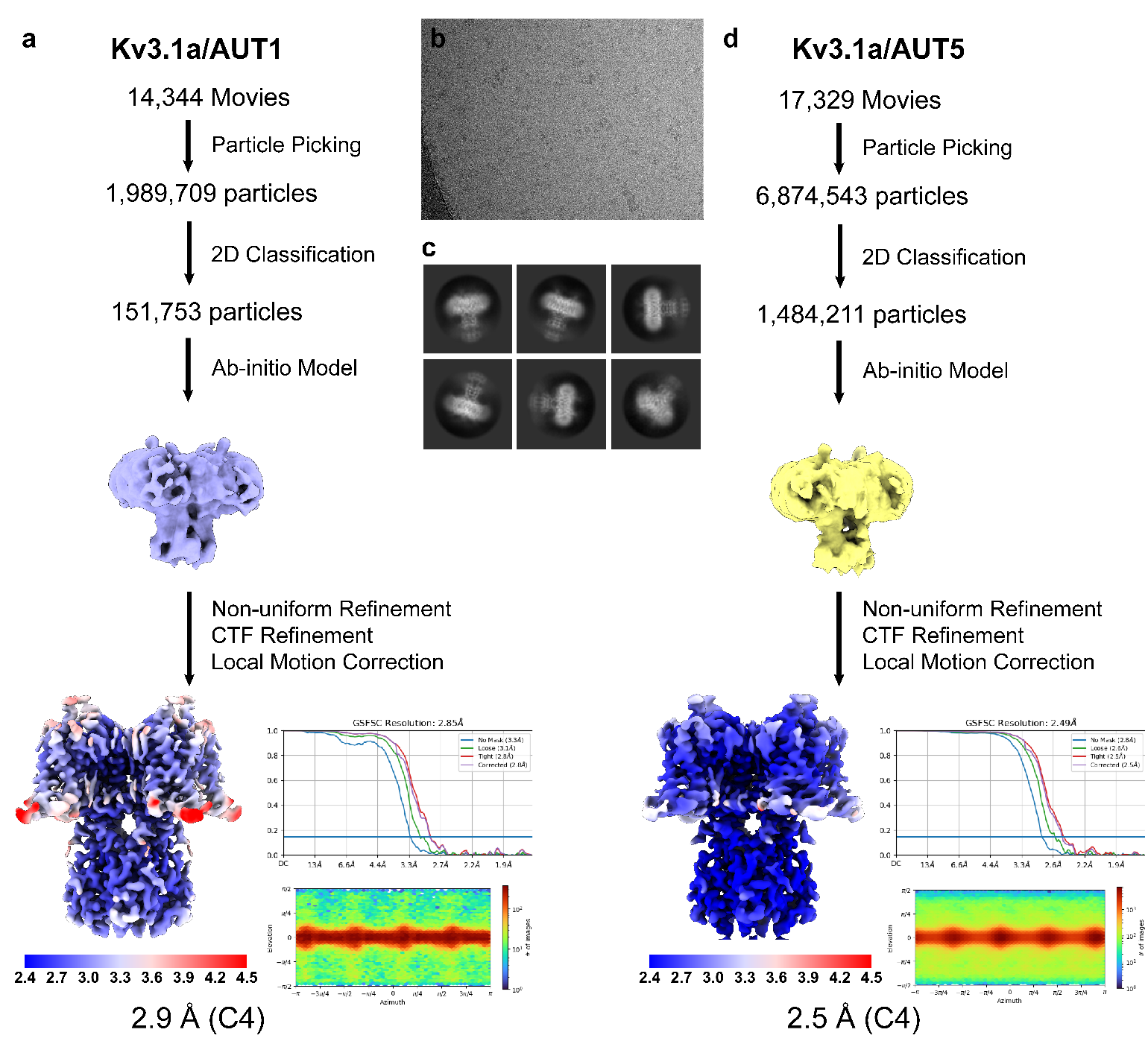
**

**Figure S6.**  **Cryo-EM data processing of the Kv3.1/AUT1 and Kv3.1/AUT5 complexes. (a)** Processing workflow for Kv3.1/AUT1 dataset. **(b)** Representative cryo-EM micrograph for Kv3.1/AUT1 dataset. **(c)** Representative 2D classes for Kv3.1/AUT1 dataset. **(d)** Processing workflow for Kv3.1/AUT5 dataset**.**

**
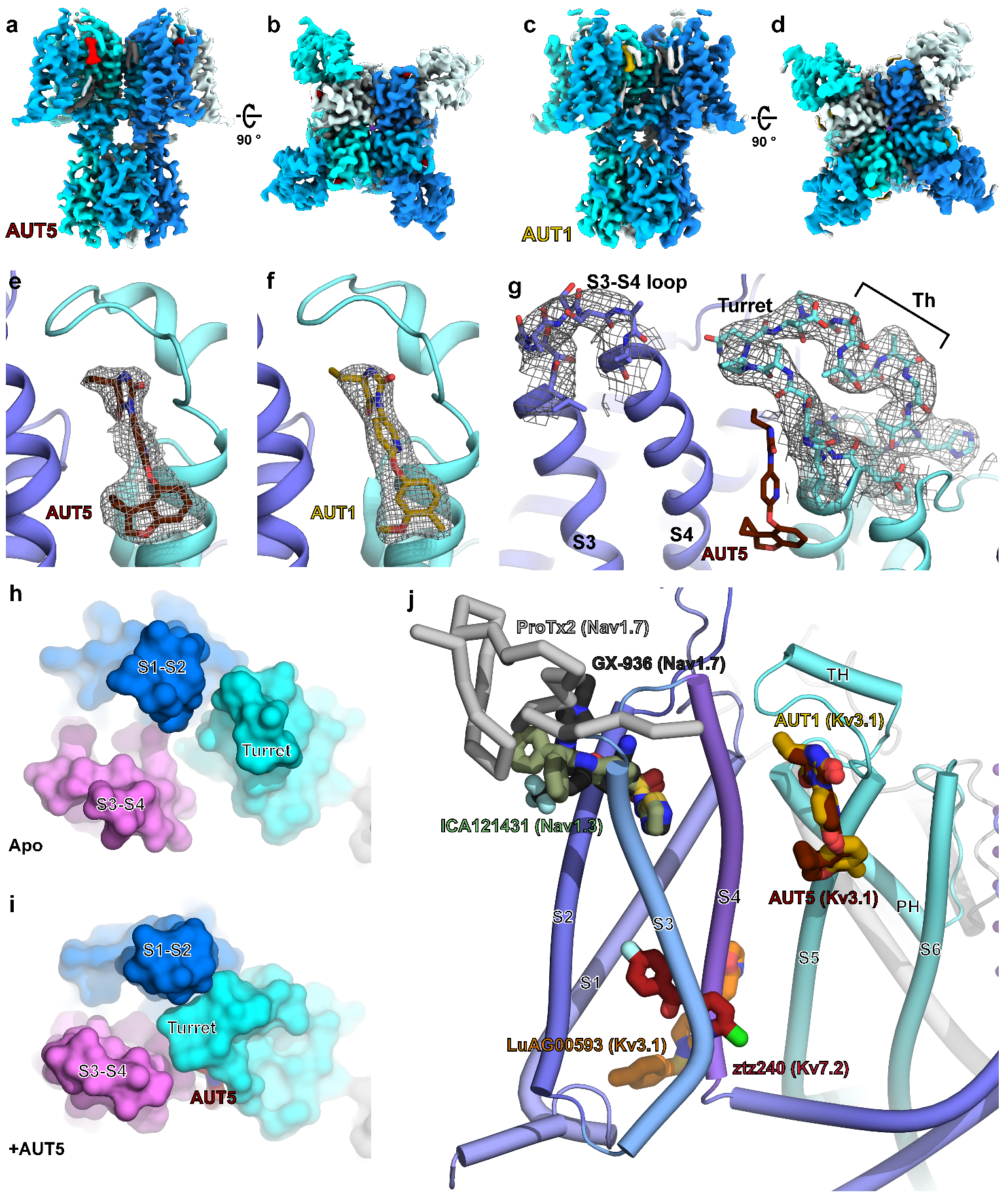
**

**Figure S7. Additional structural analysis of the Kv3.1/AUT1 and Kv3.1/AUT5 complexes. (a – b)** Cryo-EM reconstruction of Kv3.1/AUT5 complex dataset, with the map viewed from the bilayer side (a), and from the extracellular side (b)**. (c-d)** Cryo-EM reconstruction of Kv3.1/AUT1 complex dataset, with the map viewed from the bilayer side (c), and from the extracellular side (d). **(e)** Structure model of AUT5 (dark red) and its EM map feature (grey mesh). **(f)** Structure model of AUT1 (gold) and its EM map feature (grey mesh). **(g)** Structure model of S3-S4 loop (slate blue) and Turret (cyan) with their EM map features (grey mesh). **(h)** Surface representation of apo-state Kv3.1 at the AUT5-binding site. Turret (cyan) is in weak interacting distance from S1-S2 linker (blue) and makes no interaction with S3-S4 loop (magenta). **(i)** Surface representation of AUT5-bound Kv3.1a at the modulator-binding site. Turret and S3-S4 loop have made conformational changes. Turret interacts with S1-S2 linker more closely, and it is within interaction distance from S3-S4 loop. **(j)** Locations of binding sites for various VSD-modulating compounds. AUT1 (gold), AUT5 (dark red), LuAG00593 (orange, PDB ID: 7PQU) and ztz240 (red, PDB ID: 7CR1) are positive modulators, and their binding sites vary. ProTx-2 (grey, PDB ID: 6N4Q), GX-936 (black, PDB ID: 5EK0) and ICA121431 (green-grey, PDB ID: 7W7F) are negative modulators, and their binding sites are localized to S2 and S3 helices on the extracellular side.


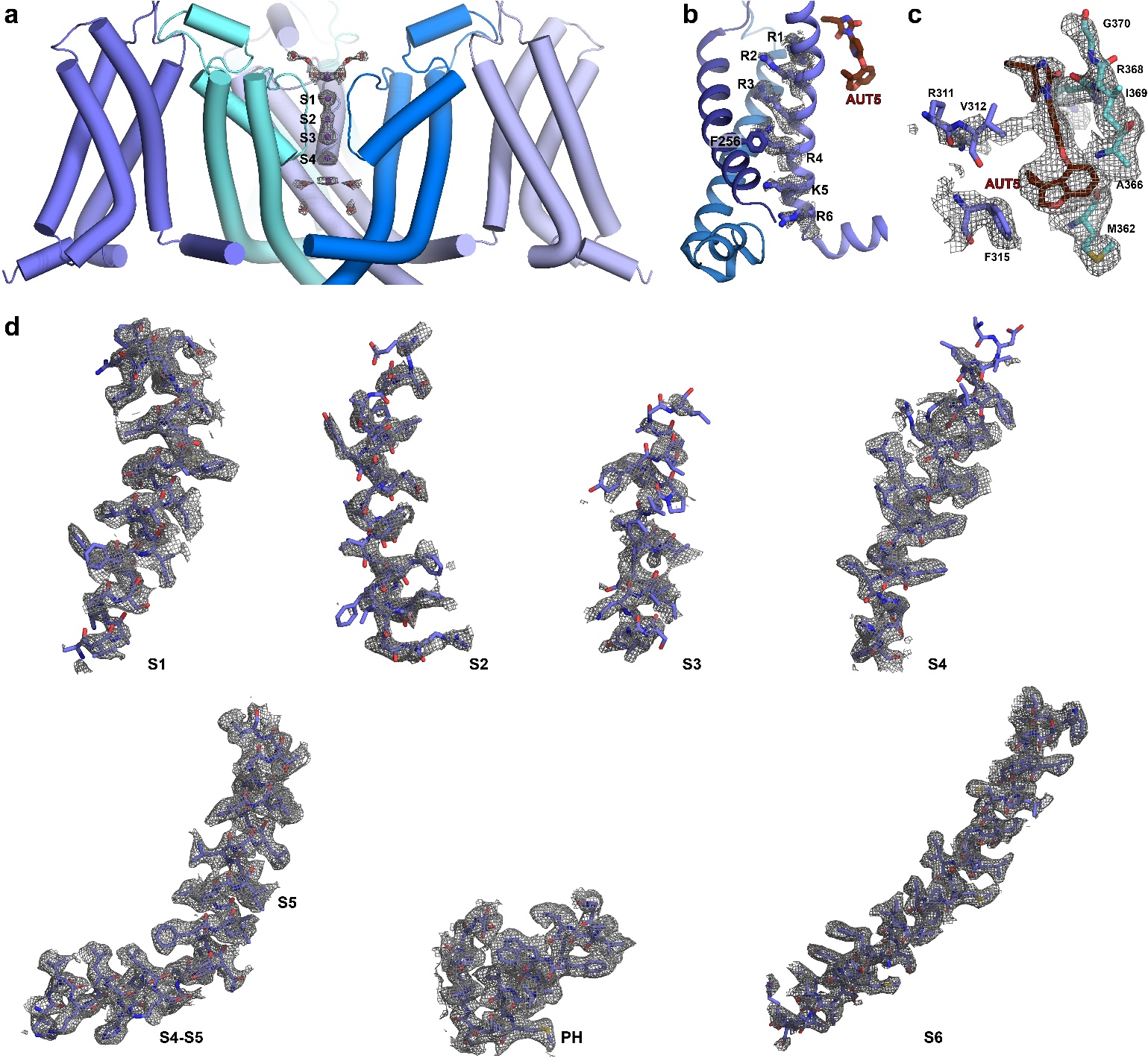


**Figure S8. Cryo-EM map and model of the Kv3.1/AUT5 complex selectivity filter and voltage sensor residues. (a)** Cartoon representation of Kv3.1 transmembrane domain. EM map clearly shows features for potassium ions in all four sites of selectivity filter (S1 – S4) as well as hydrated ions on the extracellular and cytoplasmic sides. (**b**) Cartoon representation of Kv3.1 VSD. EM map features for voltage-sensing residues (R1 – R6) are highlighted with grey mesh. (**c**) Stick representation of AUT5 and residues in its nearby environment with EM map features highlighted as grey mesh. (**d**) Stick representations of transmembrane helices (S1 - S5, PH, S6) with EM map features highlighted as grey mesh.

**a**

hKv3.2 ----------------------------MGKIENNERVILNVGGTRHETYRSTLKTLPGT 32

hKv3.1 ----------------------------MGQGDESERIVINVGGTRHQTYRSTLRTLPGT 32

hKv3.4 MISSVCVSSYRGRKSGNKPPSKTCLKEEMAKGEASEKIIINVGGTRHETYRSTLRTLPGT 60

*.: : .*::::*******:******:*****

T1-----------------------

hKv3.2 RLALLASSEPPGDCLTTAGDKLQPSPPPLSPPPRA**PPLSPGPGGCFEGGAGNCSSRGGRA** 92

hKv3.1 RLAWLAEPDAHSHFDYD------------------**-------------------------** 49

hKv3.4 RLAWLADPDGGGRPETD------------------**-----------GGGVG-------SS** 84

*** **. : .

------------------------------------------------------------

hKv3.2 **SDHPGGGR**EFFFDRHPGVFAYVLNYYRTGKLHCPADVCGPLFEEELAFWGIDETDVEPCC 152

hKv3.1 **----PRAD**EFFFDRHPGVFAHILNYYRTGKLHCPADVCGPLYEEELAFWGIDETDVEPCC 105

hKv3.4 **GSSGGGGC**EFFFDRHPGVFAYVLNYYRTGKLHCPADVCGPLFEEELTFWGIDETDVEPCC 144

. ************::*******************:****:*************

----------T1

hKv3.2 WMTYRQHRDAEEALDIFETPDLIGGD-------P----GDDEDLA--AKRLGIEDAAGLG 199

hKv3.1 WMTYRQHRDAEEALDSFGGAPLDNSADDADADGPGD-SGDGEDELEMTKRLALS----DS 160

hKv3.4 WMTYRQHRDAEEALDIFESPDGGGS-----GAGPSDEAGDDERELALQ-RLGPH--EGGA 196

*************** * . * ** * **. .

S1-----------------S1

hKv3.2 GPDGKSGRWRRLQPRMWALFEDPYSSRAARFIAFASLFFILVSITTFCLETHEAFN**IVKN** 259

hKv3.1 PDGRPGGFWRRWQPRIWALFEDPYSSRYARYVAFASLFFILVSITTFCLETHERFN**PIVN** 220

hKv3.4 GHGAGSGGCRGWQPRMWALFEDPYSSRAARVVAFASLFFILVSITTFCLETHEAFN**IDRN** 256

.* * ***:*********** ** :********************* ** *

S2----------------S2 S3--

hKv3.2 **KTEP--VINGTSVVLQYEIETDPA**LTYVEGVCVVWFTFEFLVRIVFSPNKLEFIKNLLNI 317

hKv3.1 **KTEIENVRNGTQVRYYREAETEAF**LTYIEGVCVVWFTFEFLMRVIFCPNKVEFIKNSLNI 280

hKv3.4 **VTEILRVGNITSVHFRREVETEPI**LTYIEGVCVLWFTLEFLVRIVCCPDTLDFVKNLLNI 316

** * * *.* * **: ***:*****:***:***:*:: .*:.::*:** ***

---------------S3 S4-------------------S4

hKv3.2 IDFVAILPFYLEVGLSGLSSKAAKDVLGFLRVVRFVRILRIFKLTRHFVGLRVLGHTLRA 377

hKv3.1 IDFVAILPFYLEVGLSGLSSKAAKDVLGFLRVVRFVRILRIFKLTRHFVGLRVLGHTLRA 340

hKv3.4 IDFVAILPFYLEVGLSGLSSKAARDVLGFLRVVRFVRILRIFKLTRHFVGLRVLGHTLRA 376

***********************:************************************

S5-----------------S5

hKv3.2 STNEFLLLIIFLALGVLIFATMIYYAER**VGAQPNDPSASEHTQ**FKNIPIGFWWAVVTMTT 437

hKv3.1 STNEFLLLIIFLALGVLIFATMIYYAER**IGAQPNDPSASEHTH**FKNIPIGFWWAVVTMTT 400

hKv3.4 STNEFLLLIIFLALGVLIFATMIYYAER**IGARPSDPRGNDHTD**FKNIPIGFWWAVVTMTT 436

****************************:**:*.** ..:**.*****************

S6-----------------S6

hKv3.2 LGYGDMYPQTWSGMLVGALCALAGVLTIAMPVPVIVNNFGMYYSLAMAKQKLPRKRKKHI 497

hKv3.1 LGYGDMYPQTWSGMLVGALCALAGVLTIAMPVPVIVNNFGMYYSLAMAKQKLPKKKKKHI 460

hKv3.4 LGYGDMYPKTWSGMLVGALCALAGVLTIAMPVPVIVNNFGMYYSLAMAKQKLPKKRKKHV 496

********:********************************************:*:***:

hKv3.2 PPAPQASSPTFCKTELNMACNSTQSDTCLGKDN-RLLEHNRS-----------VLSGDDS 545

hKv3.1 PRPPQLGSPNYCKSVVNSPHHSTQSDTCPLAQE-EILEINRADSKLNGEVAKAALANEDC 519

hKv3.4 PRPAQLESPMYCKSEETSPRDSTCSDTSPPAREEGMIERKRADSKQNGDANAV-LSDEEG 555

* * ** :**: . .** ***. : ::* :*: *: ::

hKv3.2 TGSEP----PLSPPERLPIRRSSTRDKNRRGETCFLLTTGDYTCASDGGIRKDNCKEVVI 601

hKv3.1 PHIDQ----ALTPDEGLPFTRSGTR---ERYGPCFLLSTGEYACPPGGGMRKDLCKESPV 572

hKv3.4 AGLTQPLASSPTPEERRALRRSTTRDRNKKAAACFLLSTGDYACADGSVRKGTFVLRDLP 615

:* * : ** ** .: ****:**:*:* . : .

hKv3.2 T-GYTQAEARSLT-------- 613

hKv3.1 IAKYMPTEAVRVT-------- 585

hKv3.4 L-QHSPEAACPPTAGTLFLPH 635

: * *

**b**

rKv1.1 YFAEAEEAES-------------------------------HFSSIP 360

rKv1.2 YFAEADERDS-------------------------------QFPSIP 362

rKv2.1 FFAEKDEDD-------------------------------TKFKSIP 361

rKv3.1 YYAERIGAQPNDPSASEH----------------------THFKNIP 388

rKv3.2 YYAERVGAQPNDPSASEH----------------------TQFKNIP 425

dShaw YYAERIQPNP-------H----------------------NDFNSIP 365

rKv4.1 FYAEKGTSKT-------------------------------NFTSIP 360

rKv5.1 YTMEQSHPE-------------------------------TLFKSIP 370

rKv6.1 YVIENEMADS------------------------------PEFTSIP 412

rKv7.1 YLAEKDAVNES---------------------------GRIEFGSYA 300

rkV8.1 YFAEQSIPD-------------------------------TTFTSVP 383

rKv9.1 YTAEEKNVG---------------------------------FDTIP 380

rKv10.1 IFDEDTKTIRNNSWLYQLALDIGTPYQFNGSGSGKWEGGPSKNSVYI 424

rKv11.1 QPHMDSHIG----WLHNLGDQIGKPYNSSGLG------GPSIKDKYV 614

rKv12.1 R-EDNSLLKWEVGWLHELGKRLESPYYGNNTLG-----GPSIRSAYI 422

KvAP IYIVEYPDPN------------------------------SSIKSVF 197

Figure S9. Sequence alignments of Kv channel amino acid sequences. (a) Full-length sequence comparisons between the human variants of Kv3.1a, Kv3.2a and Kv3.4a. Red characters highlight the T1 domain, and the transmembrane segments S1-S6. Excluding the C-terminal regions, which diverge greatly among these Kv3 channels, the bold characters highlight regions with significant divergence in the T1 domain, the S1-S2 loop and the extracellular S5-PH linker (‘turret’). Conserved putative contact sites in the AUT5 binding pocket are highlighted in cyan. (b) Sequence comparison of the turrets from representative members of the Kv channel family. Red characters highlight the unique turret regions of Kv3.1a and Kv3.2a.


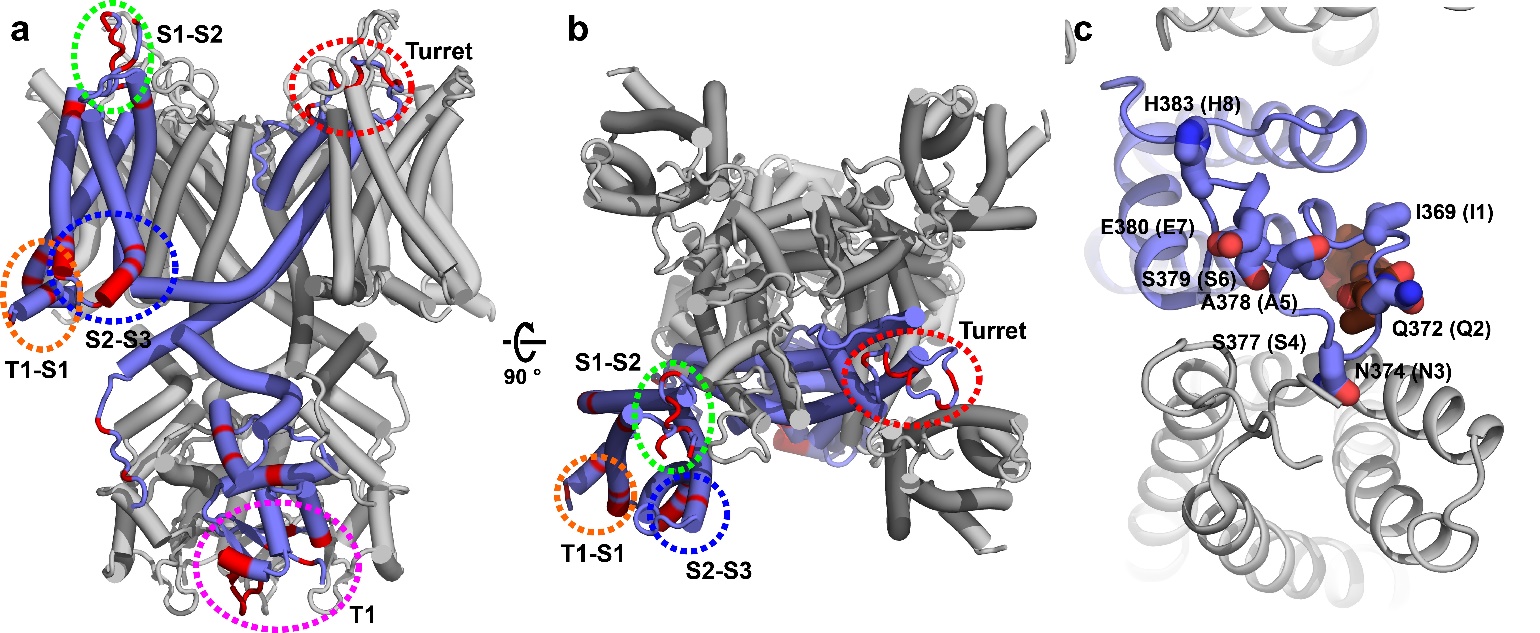


**Figure S10. Amino acid sequence divergence between Kv3.1 and Kv3.4 with structural context. (a, b)** Cartoon structure model of Kv3.1 (with AUT5) with one subunit highlighted in slate blue, viewed from the bilayer side (a) and extracellular side (b). Residues with different amino acids to Kv3.4 are highlighted in red. (**c**) Cartoon representation of Kv3.1 turret region viewed from the extracellular side. Residues with different sequences to Kv3.4 are marked with stick representations.

**
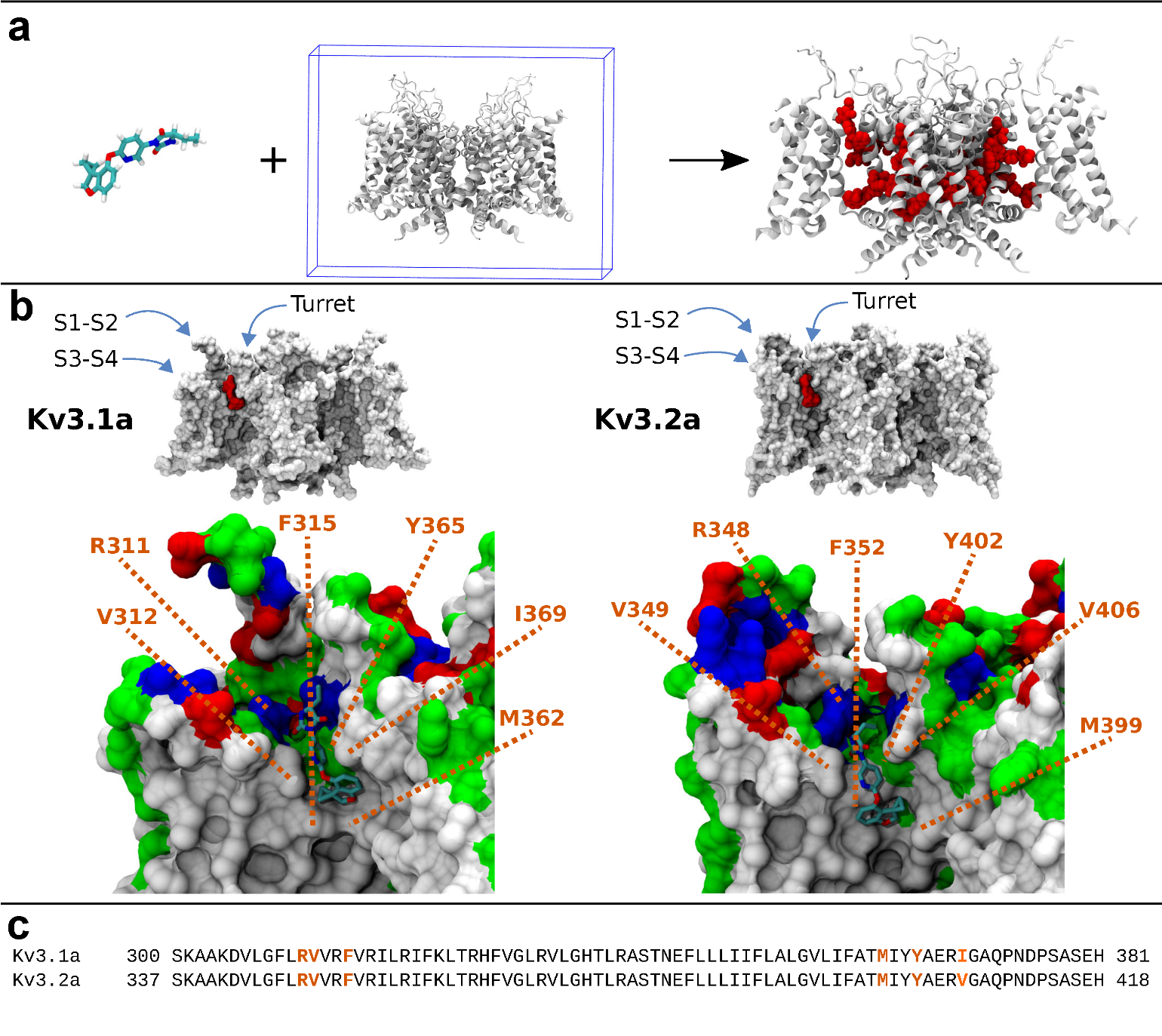
**

**Figure. S11. Blind docking calculations of AUT5 binding to Kv3.1 and Kv3.2.** (**a**) Molecular structures of AUT5, Kv3.1a (white) and docking solutions (red) across the search grid volume. (**b**) Surface representation of Kv3.1a, Kv3.2a showing the AUT5 bound molecule at a site near the turret. Inset details the bound molecule and highly conserved amino acids at the binding site. (**c**) Sequence alignment of S4-S5 regions of Kv3.1a and Kv3.2a channels.


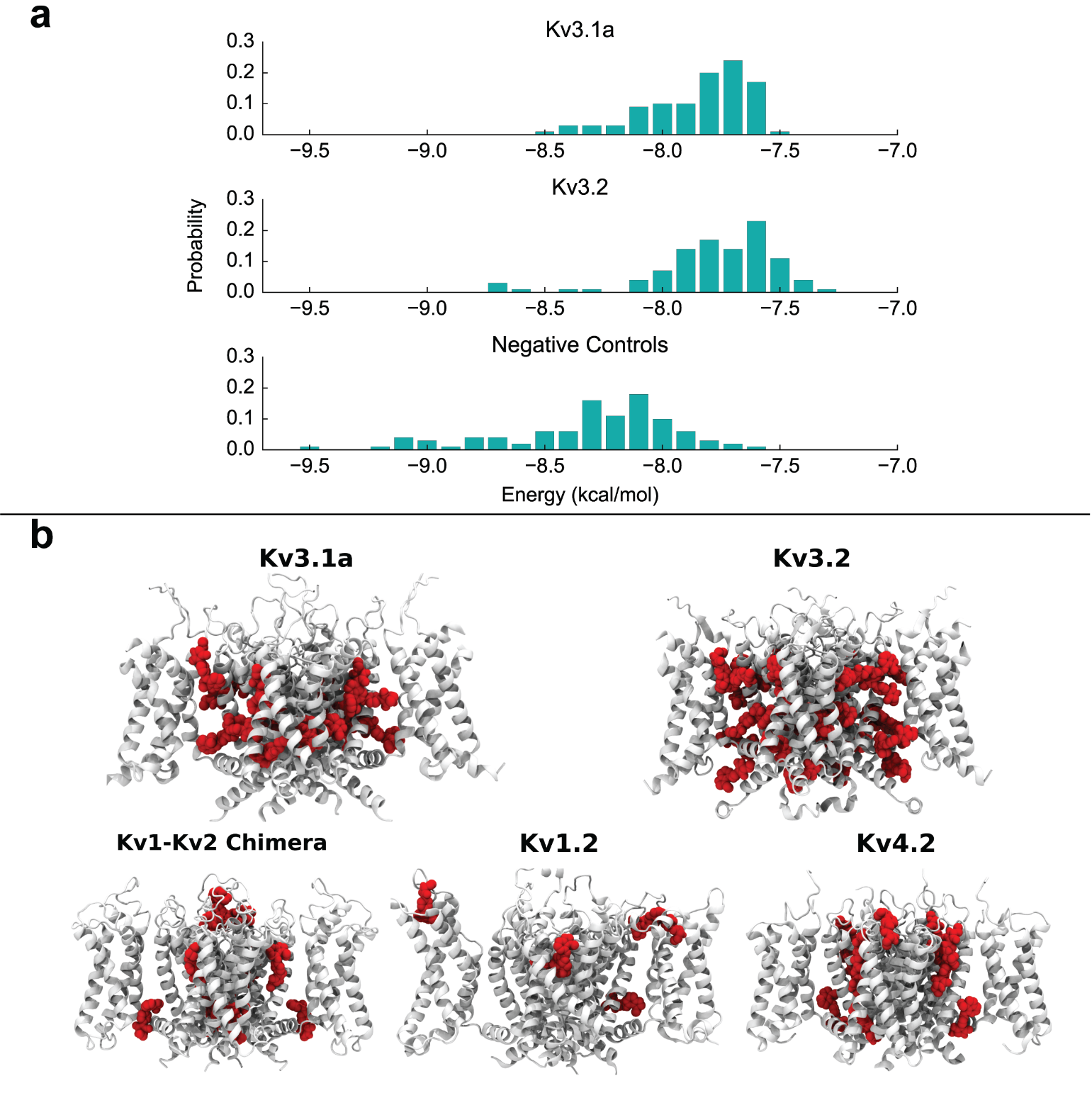


**Figure S12. Distribution of binding energy values for all suggested docking sites.** (**a**) Probabilities were based on the total number of suggested solutions from docking calculations of AUT5 with Kv3.1a, Kv3.2 or negative controls (Kv1-Kv2 Chimera, Kv1.2 and Kv4.2). Each distribution contains at least 80 independent docking solutions. (**b**) Best AUT5 docking solutions (red) within the sites determined for Kv3.1a, Kv3.2a and negative controls (Kv1-Kv2 Chimera, Kv1.2 and Kv4.2). Sites were defined by a proximity criterion and their positioning in the protein.

**
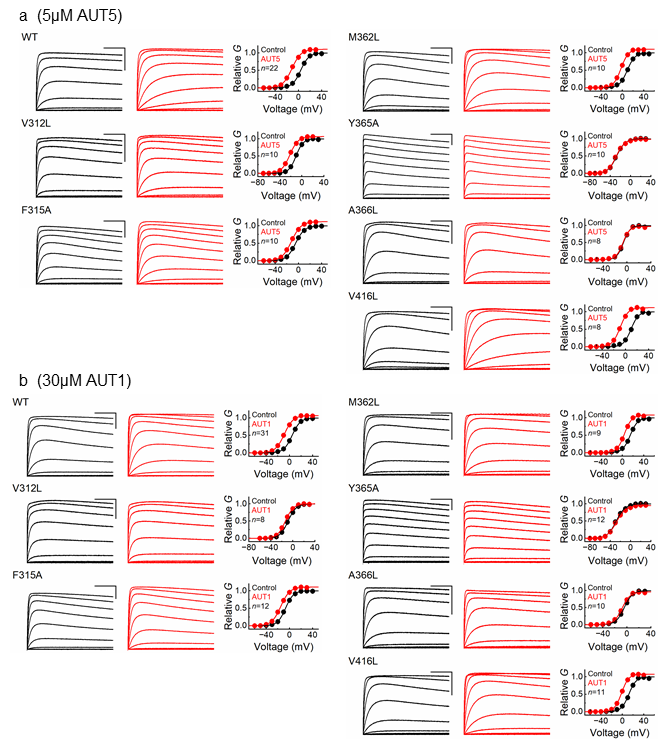
**

**Figure S13. Mutational and functional analyses of AUT5 and AUT1 binding site determinants.** (**a**) Families of Kv3.1a currents (WT and the indicated mutants) expressed in *Xenopus* oocytes, before and after exposure to 5 μM AUT5 (left and center). All current families were evoked by the voltage protocol shown on Fig. S1d and described in the corresponding legend. The corresponding aggregate analysis of the corresponding G_p_ – V_c_ curves is also shown (right), including the best-fit Boltzmann function (solid line). (**b**) Same as panel a, except that oocytes were exposed to 30 mM AUT1. SEM bars are in most instances smaller than the symbols.





**Figure S14. The effects of deleting the T1 domain and swapping the extracellular S1S2 linker on the basal gating properties and AUT5 sensitivity of Kv3.2.** (**a**) Families of whole-oocyte currents expressed by the indicated Kv3.2 mutants. 3.4x3.2 S1S2 is the Kv3.2 chimera in which the S1S2 linker from Kv3.4 is swapped for that from Kv3.2. All current families were evoked by the voltage protocol shown on Fig. S1d and described in the corresponding legend. Scale bars represent 1 μA and 100 ms. (**b**) Overlay of G_p_ – V_c_ curves obtained under basal conditions. (**c**) and (**d**) Overlay of G_p_ – V_c_ curves obtained from current recordings in the absence and presence of 2 μM AUT5. Solid lines in (b), (c) and (d) are the best fits of the 1^st^-order Boltzmann equation (Methods). The table below summarizes the V_0.5_ changes induced by the mutations and application to AUT5.

| Kv3.2 | *n* | *V*_0.5_ (Control, mV) | *V*_0.5_ (2 μM AUT5, mV) | Δ*V*_0.5_ (mV) | *P* (*vs*. WT) |
| --- | --- | --- | --- | --- | --- |
| WT | 74 | -2.7±0.5 | -29.2±1.1 | -26.5±0.9 |  |
| ΔT1 | 5 | 4.9±1.2 | -21.5±2.9 | -26.4±2.2 | 0.88792 |
| 3.4x3.2 S1S2 | 8 | 1.5±1.3 | -16.5±1.5 | -17.9±1.3 | 7x10^-4^ |





**Figure S15. The effect of various Kv3.2 turret mutations on voltage-dependent gating under basal conditions.** (**a**) Overlay of G_p_ – V_c_ curves for Kv3.2 WT and the turret mutations indicated on the graph. The positions of the mutations are as indicated on Fig. 6 and Table S2. Solid lines are the best fits of the 1^st^ order Boltzmann equation. (**b**) Scatter plots comparing the V_0.5_ of the G_p_ – V_c_ curves. Each symbol is a measurement from an individual oocyte, and the bars indicate the mean values. The red dashed line is shown as the WT reference value of the V_0.5_. Sample size is indicted in Fig 8.





**

**

**Figure S16. The effect of various Kv3.4 turret mutations on voltage-dependent gating under basal conditions.** (**a**) Overlay of G_p_ – V_c_ curves for Kv3.4 WT and the turret mutations indicated on the graph. Solid lines are the best fits of the 1^st^ order Boltzmann equation (Methods). (**b**) Scatter plots comparing the V_0.5_ of the G_p_ – V_c_ curves. Each symbol is a measurement from an individual oocyte, and the bars indicate the mean values. The red dashed line is shown as the WT reference value of the V_0.5_. (**c**) Overlay of G_p_ – V_c_ curves for Kv3.4 WT and the turret mutations indicated on the graph. Solid lines are the best fits of the 1^st^ order Boltzmann equation. (**d**) Scatter plots comparing the V_0.5_ of the G_p_ – V_c_ curves. Other details as indicated above for (b). a and b are experiments without PMA. c and d are experiments with PMA. Number of oocytes (a and b): 28 (WT), 10 (3.2x3.4 turret), 10 (Δturret). Sample size of c and d is indicted in Fig 8. The positions of the mutations are as indicated on Fig. 6 and Table S2.





**Figure S17. The effects of AUT5 following elimination of fast Kv3.4 inactivation by activation of PKC.** (**a**) Families of WT Kv3.4 whole-oocyte currents evoked by the pulse protocol shown on Fig. S1d, before and after bath application to 50 nM PMA to activate PKC (Methods) and following application to 2 μM AUT5. PMA remained present during the application of AUT5. Scale bars represent 1 μA and 100 ms. (**b**) Time course of the elimination of fast Kv3.4 inactivation upon bath application to 50 nM PMA. The currents evoked by a 400 ms test pulse to +20 mV from a holding potential of -100 mV. The interval between trace 0 and 1 is 20 seconds. From trace 1 to trace 8, the interval between two adjacent traces is 1 minute. (**c**) and (**d**) Overlay of G_p_ – V_c_ curves under the conditions indicated on the graph. Solid lines are the best fits of the 1^st^ order Boltzmann equation. (**e**) Scatter plots of the changes in the best fit parameters. Each pair of connected symbols (before and after 2 μM AUT5) represents a measurement from an individual oocyte, and the bar represents the mean. The two-sided paired Student t-test was used to evaluate the significance of the changes. P values are indicated above each plot.





























**Figure S18. Gating properties of Kv3.2 mutants before and after bath application of 2 μM AUT5.** (**a**) – (**i**) (top, left and center) Families of mutant Kv3.2 whole-oocyte currents evoked by the pulse protocol shown on Fig. S1d, before (left, black) and after (center, red) bath application of 2 μM AUT5. Scale bars represent 1 μA and 100 ms. (**a**) – (**i**) (top, right) Overlay of G_p_ – V_c_ curves under the conditions indicated on the graph. Solid lines are the best fits of the 1^st^ order Boltzmann equation. (**a**) – (**i**) (bottom) Scatter plots of the changes in the best fit parameters V_0.5_, G_max_ and z. Each pair of connected symbols (before and after 2 μM AUT5) represents a measurement from an individual oocyte, and the bar represents the mean. The two-sided paired Student t-test was used to evaluate the significance of the changes. P values are indicated above each plot.













**Figure S19. Gating properties of Kv3.4 mutants before and after bath application of 2 μM AUT5 and in the presence of PMA.** (**a**) – (**e**) (top, left and center) Families of mutant Kv3.4 whole-oocyte currents evoked by the pulse protocol shown on Fig. S1d, before (left, blue) and after (center, red) bath application of 2 μM AUT5. Scale bars represent 1 μA and 100 ms. (**a**) – (**e**) (top, right) Overlay of G_p_ – V_c_ curves under the conditions indicated on the graph. Solid lines are the best fits of the 1^st^ order Boltzmann equation. (**a**) – (**e**) (bottom) Scatter plots of the changes in the best fit parameters V_0.5_, G_max_ and z. Each pair of connected symbols (before and after 2 μM AUT5) represents a measurement from an individual oocyte, and the bar represents the mean. The two-sided paired Student t-test was used to evaluate the significance of the changes. P values are indicated above each plot. All experiments are with 50 nM PMA.

**
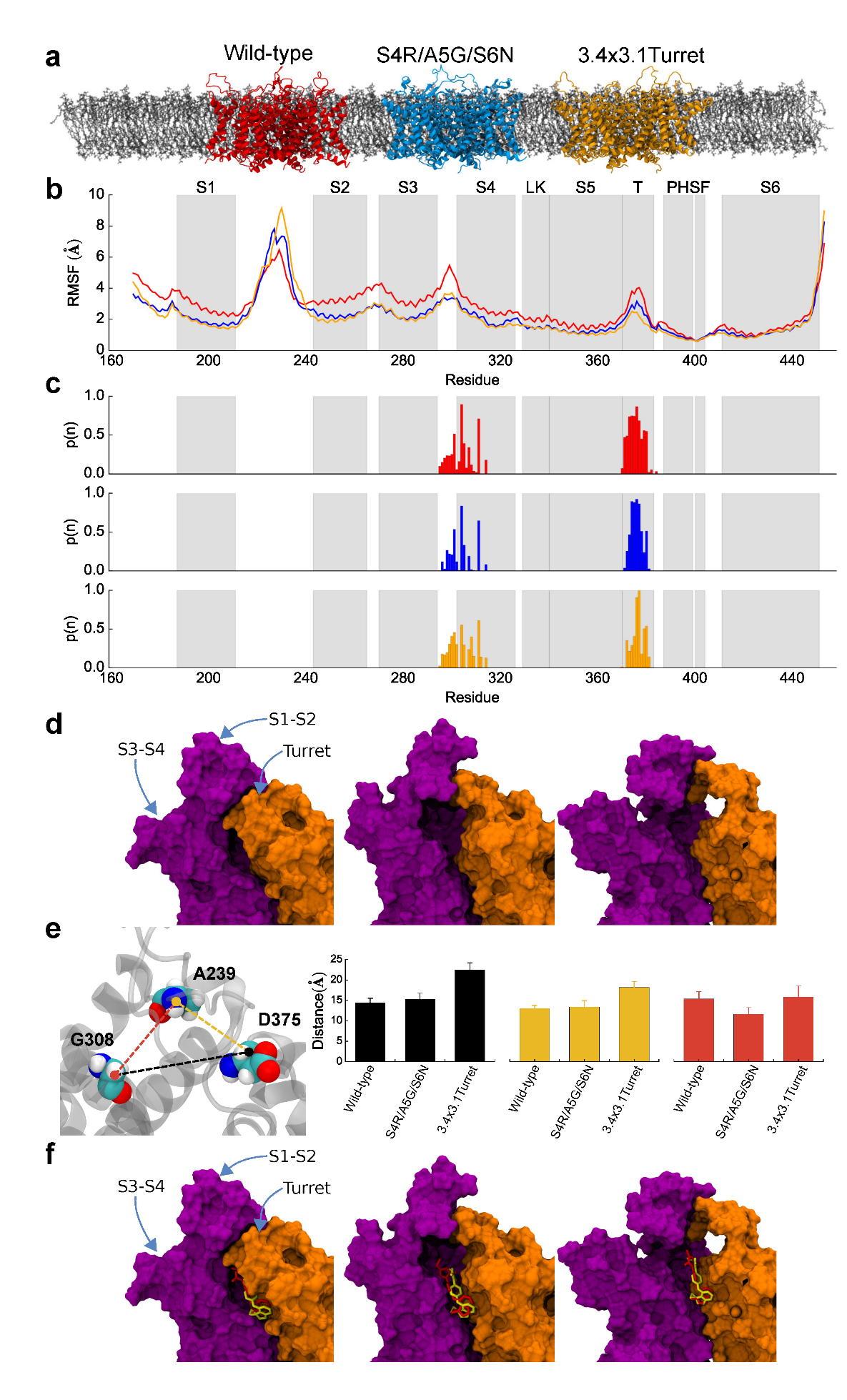
**

**Figure S20. Turret mutations disrupt key interactions with the VSD without altering the AUT5 binding site.** (**a**) Steady-state structures of Kv3.1a (red), 3.1x3.4Turret (orange) and S4R/A5G/S6N (blue) in the phospholipid bilayer. (**b**) Root-mean-square fluctuations. Transmembrane helices (S1 to S6), linker (LK), turret (T), pore helix (PH) and selectivity filter (SF) are indicated. (**c**) Probability of amino-acid contacts of the S1-S2 loop. Amino-acid contacts were defined for a cutoff distance of 5 Å. The cumulative probabilities along channel residues $\sum p\left( n \right)$ are: 11.11 (WT), 9.40 (3.1x3.4Turret) and 9.32 (S4R/A5G/S6N). (**d**) Molecular representations of S1-S2 loop interactions with the voltage-sensor (purple) and pore (orange). (**e**) Separation distances between the turret and the voltage-sensor loops S1-S2 and S3-S4 for each of the channel structures in (d). Separation distances were calculated between C_α_ atoms of the report amino acids A239, G308 and D375 respectively located in the S1-S2 loop, S3-S4 loop and turret. (**f**) Docking solutions (red) that best reproduce the cryo-EM bound configuration (yellow) of AUT5.

**Figure S21. Comparison of the cryo-EM structures of the Kv3.1/AUT5 and Kv3.1/Compound-4 complexes.** Dark blue - VSD of subunit A in Kv3.1/AUT5; cyan - pore domain of subunit B in Kv3.1/AUT5; brown - AUT5 compound; purple - VSD of subunit A in Kv3.1/Compound-4; pink - pore domain of subunit B in Kv3.1/Compound-4; orange - Compound 4. Compound 4 structure is from PDB ID: 8F1C. (**a**) Comparison of the two structures at the modulator-binding site show similar binding modes for the two compounds. Residues surrounding the compounds are also similar between the two structures, with their labelled residues having root-mean-squared-distance (R.M.S.D.) value of 0.8 Å. (**b**) Comparison of the two structures at the Turret-VSD interface seen from the extracellular side show the two structures in similar states. Turret loop in both structures have similar conformational changes including its shift toward the modulators and formation of turret helix (TH). R.M.S.D. for the turret between the two structures is 0.8 Å.

**Table S1.** **Cryo-EM data collection, processing parameters and model refinement statistics for Kv3.1a datasets collected with AUT1 and AUT5-incubated conditions.**

|  | **Kv3.1 with AUT1** | **Kv3.1 with AUT5** |
| --- | --- | --- |
| **Data Collection Setting** |  |  |
| Microscope | Titan Krios (eBIC, UK) | Titan Krios (eBIC, UK) |
| Detector | K3 | K3 |
| Voltage (kV) | 300 | 300 |
| Collection Mode | Counting  (superresolution, 2x binned) | Counting  (superresolution, 2x binned) |
| Electron Exposure (e/Å^2^) | 50 | 50 |
| Number of Frames | 50 | 50 |
| Nominal Magnification | 105,000 | 105,000 |
| Pixel Size (Å/pix) | 0.831 | 0.825 |
| Defocus Range (µm; steps) | -0.8 to -2.0 (0.2) | -1.0 to -2.4 (0.2) |
| Number of Movies | 14,344 | 17,329 |
|  |  |  |
| **Data Processing** |  |  |
| Initial Number of Particles | 1,989,709 | 6,874,543 |
| Number of Particles after 2D symmetry | 151,753 | 1,484,211 |
| Number of Particles Used for 3D Refinement | 151,753 | 1,484,211 |
| Symmetry | C4 | C4 |
| Map Resolution  (Å; FSC threshold = 0.143) | 2.9 | 2.5 |
| Map Sharpening B-factor (Å^2^) | -113.3 | -122.0 |
|  |  |  |
| **Refinement Parameters** |  |  |
| Model Resolution  (Å; FSC threshold = 0.5) | 2.9 | 2.7 |
| Map/model Cross-correlation | 0.87 | 0.76 |
| Q-Score | 0.5510 | 0.5550 |
|  |  |  |
| **Model Composition** |  |  |
| Non-hydrogen Atoms | 12,829 | 12,806 |
| Protein Residues | 1,580 | 1,580 |
| Ligands | 21 | 22 |
| **R.M.S.D** |  |  |
| Bond Lengths | 0.004 | 0.003 |
| Bond Angles | 0.592 | 0.575 |
| **Validation** |  |  |
| Molprobity Score | 1.66 | 1.31 |
| Clash Score | 6.18 | 6.23 |
| Rotamer Outliers (%) | 1.51 | 0.49 |
| **Ramachandran Plot** |  |  |
| Disallowed (%) | 0.00 | 0.00 |
| Allowed (%) | 3.08 | 2.31 |
| Favoured (%) | 96.92 | 97.69 |
|  |  |  |
| **EMDB Code** | EMD-18659 | EMD-18660 |
| **PDB Code** | 8QUC | 8QUD |

**Table S2. Definitions of mutations**

|  | Definition |
| --- | --- |
| Kv3.2 |  |
| N3S | N411S |
| S4R | S414R |
| A5G | A415G |
| S6N | S416N |
| N3S/S4R | N411S/S414R |
| N3S/A5G | N411S/A415G |
| N3S/S6N | N411S/S416N |
| S4R/A5G | S414R/A415G |
| S4R/A5G/S6N | S414R/A415G/S416N |
| 3.4x3.2Turret | V406I/Q409R/N411S/S414R/A415G/S416N/E417D/Q420D |
| ΔTurret | deletion of N411 - E417 (NDPSASE) |
| ΔT1 | deletion aa 2 - 215 |
| 3.4x3.2 S1S2 | Kv3.2 S1S2 linker (aa 257-281) replaced with Kv3.4’s (aa 254-280) |
| Kv3.4 |  |
| S3N | S410N |
| R4S | R413S |
| G5A | G414A |
| N6S | N415S |
| S3N/N6S | S410N/N415S |
| 3.2x3.4Turret | S5S6 linker (aa 405 - 419) replaced with hKv3.2 (aa 406 - 420) |
| ΔTurret | deletion of N410 - E416 (SDPRGND) |

**Table S3. Primers used for mutagenesis of Kv3.2 and Kv3.4**

| Name | Sequence |
| --- | --- |
| Kv3.2 |  |
| N3S Fwd | 5'-GAGTGGGAGCTCAACCTAGCGACCCTTCAG-3' |
| Rev | 5'-CTGAAGGGTCGCTAGGTTGAGCTCCCACTC -3' |
| S4R Fwd | 5’-CAACCTAACGACCCTCGAGCTAGTGAGCACACAC-3’ |
| Rev | 5’-CAACCTAACGACCCTCGAGCTAGTGAGCACACAC-3’ |
| A5G Fwd | 5'-CCTAACGACCCTTCAGGTAGTGAGCACACACAG -3' |
| Rev | 5'-CTGTGTGTGCTCACTACCTGAAGGGTCGTTAGG -3' |
| S6N Fwd | 5'- CAACCTAACGACCCTTCAGCTAATGAGCACACACA-3' |
| Rev | 5'- TGTGTGTGCTCATTAGCTGAAGGGTCGTTAGGTTG-3' |
| N3S/S4R Fwd | 5’-5'- GAGCTCAACCTAGCGACCCTCGAGCTAGTGAGCA-3'-3’ |
| Rev | 5’-5'- TGCTCACTAGCTCGAGGGTCGCTAGGTTGAGCTC-3'-3’ |
| N3S/A5G | template = A5G |
| Fwd | 5'-GAGTGGGAGCTCAACCTAGCGACCCTTCAG-3' |
| Rev | 5'-CTGAAGGGTCGCTAGGTTGAGCTCCCACTC -3' |
| N3S/S6N | template = S6N |
| Fwd | 5'-GAGTGGGAGCTCAACCTAGCGACCCTTCAG-3' |
| Rev | 5'-CTGAAGGGTCGCTAGGTTGAGCTCCCACTC -3' |
| S4R/A5G | template = S4R |
| Fwd | 5’-CCTAACGACCCTCGAGGTAGTGAGCACACACAG-3’ |
| Rev | 5’-CTGTGTGTGCTCACTACCTCGAGGGTCGTTAGG-3’ |
| S4R/A5G/S6N | template = S4R/A5G |
| Fwd | 5’-CTAACGACCCTCGAGGTAATGAGCACACACAGTTC-3’ |
| Rev | 5’-GAACTGTGTGTGCTCATTACCTCGAGGGTCGTTAG-3’ |
| 3.4x3.2Turret | template = S4R, 7 more steps |
|  | S414R + A415G + S416N + E417D + Q409R + N411S + V406I + Q420D |
| A415G Fwd | 5’-CCTAACGACCCTCGAGGTAGTGAGCACACACAG-3’ |
| Rev | 5’-CTGTGTGTGCTCACTACCTCGAGGGTCGTTAGG-3’ |
| S416N Fwd | 5’-CTAACGACCCTCGAGGTAATGAGCACACACAGTTC-3’ |
| Rev | 5’-GAACTGTGTGTGCTCATTACCTCGAGGGTCGTTAG-3’ |
| E417D Fwd | 5’-CGACCCTCGAGGTAATGACCACACACAGTTCAAAAAC-3’ |
| Rev | 5’-GTTTTTGAACTGTGTGTGGTCATTACCTCGAGGGTCG-3’ |
| Q409R Fwd | 5'- GAG AGT GGG AGC TCG ACC TAA CGA CCC TCG -3' |
| Rev | 5'- CGA GGG TCG TTA GGT CGA GCT CCC ACT CTC -3' |
| N411S Fwd | 5'- GTG GGA GCT CGA CCT AGC GAC CCT CGA GGT AAT G -3' |
| Rev | 5'- CAT TAC CTC GAG GGT CGC TAG GTC GAG CTC CCA C -3' |
| V406I Fwd | 5’-C TAC TAT GCC GAG AGA ATC GGA GCT CGA CCT AGC G-3’ |
| Rev | 5'- CGC TAG GTC GAG CTC CGA TTC TCT CGG CAT AGT AG -3' |
| Q420D Fwd | 5'- CGA GGT AAT GAC CAC ACA GAC TTC AAA AAC ATT CCC -3' |
| Rev | 5'- GGG AAT GTT TTT GAA GTC TGT GTG GTC ATT ACC TCG -3' |
| ΔTurret Fwd | 5'-GAGAGTGGGAGCTCAACCT CACACACAGTTCAAAAAC-3' |
| Rev | 5'-GTTTTTGAACTGTGTGTG AGGTTGAGCTCCCACTCTC-3' |
| ΔT1 | 2 steps |
| M1A Fwd | 5'- CGA ATT CCT GCA GCC CGG CAG GCA AGA TCG AGA AC -3' |
| Rev | 5'- GTT CTC GAT CTT GCC TGC CGG GCT GCA GGA ATT CG -3' |
| M154A Fwd | 5'- GAG CCC TGC TGC TGG GCG ACC TAC CGG CAG -3' |
| Rev | 5'- CTG CCG GTA GGT CGC CCA GCA GCA GGG CTC -3' |
| 3.4x3.2 S1S2 | vector = Kv3.2, insert = Kv3.4 |
| vector Fwd | 5’GCGGGAGGTAGAGACAGAGCCCATCTTGACGTATGTAGAAGGAGTGTGTG-3’ |
| Rev | 5’-GGAGGATCTCTGTCACGTTGCGGTCAATATTGAAAGCTTCATGTGTTTCC-3’ |
| insert Fwd | 5’-GGAAACACATGAAGCTTTCAATATTGACCGCAACGTGACAGAGATCCTCC-3’ |
| Rev | 5’-CACACACTCCTTCTACATACGTCAAGATGGGCTCTGTCTCTACCTCCCGC-3’ |
| Kv3.4 |  |
| S3N Fwd | 5'-GGGGCCAGGCCCAACGACCCTCGGGG-3' |
| Rev | 5'-CCCCGAGGGTCGTTGGGCCTGGCCCC-3' |
| R4S Fwd | 5'-GCCAGGCCCTCCGACCCTTCAGGTAATGACCACA-3' |
| Rev | 5'-TGTGGTCATTACCTGAAGGGTCGGAGGGCCTGGC-3' |
| G5A Fwd | 5'-TCCGACCCTCGGGCTAATGACCACACC-3' |
| Rev | 5'-GGTGTGGTCATTAGCCCGAGGGTCGGA-3' |
| N6S Fwd | 5'-CGACCCTCGGGGTAGTGACCACACCGA-3' |
| Rev | 5'-TCGGTGTGGTCACTACCCCGAGGGTCG-3' |
| S3N/N6S | template = N6S |
| Fwd | 5'-GGGGCCAGGCCCAACGACCCTCGGGG-3' |
| Rev | 5'-CCCCGAGGGTCGTTGGGCCTGGCCCC-3' |
| 3.2x3.4Turret | vector = Kv3.4, insert = Kv3.2 |
| vector Fwd | 5'- CAC AGT TCA AAA ACA TTC CCA TTG GCT TCT GGT GGG CT -3' |
| Rev | 5'- AGC CCA CCA GAA GCC AAT GGG AAT GTT TTT GAA CTG TG -3' |
| insert Fwd | 5'- TAC TAC GCT GAG CGC GTG GGA GCT CAA CCT AAC GA -3' |
| Rev | 5'- TCG TTA GGT TGA GCT CCC ACG CGC TCA GCG TAG TA -3' |
| ΔTurret Fwd | 5'-ggggccaggccc cacaccgacttc-3' |
| Rev | 5'-gaagtcggtgtg gggcctggcccc-3' |
| Kv3.1 |  |
| V312L | Prepared by GenScript |
| F315A | Prepared by GenScript |
| M362L | Prepared by GenScript |
| Y365A | Prepared by GenScript |
| A366L | Prepared by GenScript |
| V416L | Prepared by GenScript |

**Table S4. Summary of AUT5- or AUT1-induced changes in the gating parameters derived from the voltage-clamp analysis of the Kv3.1a binding site mutants.**

| **[AUT5] = 5 μM** | **N** | **ΔV_0.5_**  **(mV)** | **P*** | **Δz**  **(e_0_)** | **P*** | **ΔG_max_**  **(%)** | **P*** |
| --- | --- | --- | --- | --- | --- | --- | --- |
| Kv3.1 WT | 22 | -15.0±0.4 | 2.0x10^-20^ | -0.53±0.05 | 6.5x10^-10^ | 11.0±0.7 | 5.3x10^-8^ |
| Kv3.1 V312L | 10 | -11.3±0.3 | 1.5x10^-11^ | -0.64±0.04 | 2.1x10^-8^ | 6.6±0.7 | 3.0x10^-5^ |
| Kv3.1 F315A | 10 | -7.0±0.7 | 2.7x10^-6^ | -0.27±0.07 | 2.7x10^-3^ | 12.5±1.0 | 3.2x10^-4^ |
| Kv3.1 M362L | 10 | -11.7±0.7 | 6.3x10^-8^ | 0.06±0.15 | 0.6858 | 10.3±1.1 | 3.0x10^-4^ |
| Kv3.1 Y365A | 10 | -0.8±0.4 | 0.0780 | 0.07±0.02 | 0.0059 | -1.4±0.4 | 0.0036 |
| Kv3.1 A366L | 8 | -1.0±0.5 | 0.0705 | 0.01±0.05 | 0.8863 | -1.9±1.0 | 0.1138 |
| Kv3.1 V416L | 8 | -18.5±0.9 | 1.2x10^-7^ | -0.31±0.11 | 0.0238 | 11.9±0.9 | 2.5x10^-4^ |

| **[AUT1] = 30 μM** | **N** | **ΔV_0.5_**  **(mV)** | **P*** | **Δz**  **(e_0_)** | **P*** | **ΔG_max_**  **(%)** | **P*** |
| --- | --- | --- | --- | --- | --- | --- | --- |
| Kv3.2 WT | 11 | -15.9±1.2 | 1.0x10^-7^ | -0.62±0.07 | 5.3x10^-6^ | -0.7±0.8 | 0.4039 |
| Kv3.1 WT | 31 | -13.1±0.8 | 4.7x10^-17^ | -0.28±0.04 | 2.9x10^-7^ | 8.2±0.9 | 3.8x10^-7^ |
| Kv3.1 V312L | 8 | -5.0±1.0 | 0.0021 | -0.03±0.07 | 0.6325 | 0.3±1.1 | 0.3056 |
| Kv3.1 F315A | 12 | -8.2±0.9 | 1.4x10^-6^ | -0.24±0.04 | 7.1x10^-5^ | 10.6±0.8 | 3.3x10^-6^ |
| Kv3.1 M362L | 9 | -10.3±0.9 | 3.5x10^-6^ | 0.07±0.16 | 0.6778 | 9.9±2.2 | 0.0013 |
| Kv3.1 Y365A | 12 | 0.5±0.9 | 0.6114 | -0.03±0.04 | 0.4178 | -4.6±1.4 | 0.0251 |
| Kv3.1 A366L | 10 | -3.7±0.5 | 2.0x10^-5^ | -0.08±0.07 | 0.2923 | -3.9±1.1 | 0.0122 |
| Kv3.1 V416L | 11 | -13.4±0.8 | 8.2x10^-9^ | 0.17±0.17 | 0.3474 | 8.3±0.9 | 7.7x10^-6^ |

***** Two-tail t-Student test, comparing before and after exposure to the compounds.

**Table S5. Reagents**

| Name | Cat Number | Vendor |
| --- | --- | --- |
| AUT1 |  | Autifony Therapeutics, Ltd. (Stevenage, UK) |
| AUT5 |  | Autifony Therapeutics, Ltd. (Stevenage, UK) |
| DMSO | D128-500 | Fisher |
| Collagenase A | 10103586001 | Millipore-Sigma |
| DMEM | D5671 | Millipore-Sigma |
| L-Glutamine | G7513 | Millipore-Sigma |
| Tetracycline | T7660 | Millipore-Sigma |
| FBS | 631107 | Clontech |
| Blasticidin | ant-bl-05 | Invivogen |
| Geneticin | 10131-027 | Gibco |
| Penicillin/Streptomycin | 15070-063 | Gibco |

**Table S6. Best-fit parameters of the G_p_-V_c_ curves for Kv3.2 shown on Fig. 1b**.

|  | *V*_0.5_ (mV)* | *G*_max_ (mS)* | *z* (e_0_)* |
| --- | --- | --- | --- |
| Control | -3.7±0.9 | 0.0249±0.0022 | 4.2±0.1 |
| 2 µM AUT5 | -27.2±1.6 | 0.0217±0.0019 | 3.2±0.1 |
| Washout | -9.5±1.2 | 0.0243±0.0025 | 3.3±0.1 |

* All values are means±SEM.

**Supplementary Results and Discussion**

**The unique AUT5 binding site of Kv3 channels revealed by blind docking calculations.**

To add support to the cryo-EM structure of the protein-ligand complex, we leveraged the recently solved cryo-EM structure of the Kv3.1a channel [^1^](#_ENREF_1) and used blind docking calculations to determine the most likely location of the AUT5 binding site (Methods). These calculations and clustering analysis suggested 17 independent binding sites (Fig. S11), distributed over the transmembrane region of Kv3.1a with binding energies ranging from -8.5 to -7.5 kcal/mol (Fig. S12). Across the space of docking solutions, only site 4 was located near the turret, and mainly occupying the S4-S5 interface, as shown in Fig. S11b. In site 4, the configuration of AUT5 closely resembles the pose of the compound in the cryo-EM bound structure. The imidazolidine moiety faces polar amino acids and the benzofuran group is buried within the protein-membrane interface, making close amino acid contacts with G308, R311, V312, F315, M362, Y365, A366, R368 and I369.

Given that AUT5 acts similarly on the Kv3.1 and Kv3.2 (Fig. S1), we also investigated the interaction of this compound with a structural ColabFold [^2^](#_ENREF_2) generated model of Kv3.2. The same docking and analysis protocols described above suggested 28 transmembrane sites with binding energies ranging from -8.7 to -7.3 kcal/mol (Fig. S12). AUT5 was found to occupy a unique site near the turret, making contacts with R348, V349, F352, I355, M399, Y402 and V406. With a root mean square deviation of 5.86 Å, including rotation and translation of the molecule within the binding site, docking solutions of AUT5 are structurally similar between Kv3.1 and Kv3.2, further corroborating that the binding pocket located near the turret is critical in the mechanism of action. Considering the high selectivity of the compound (Fig. S1), we also conducted blind docking calculations with AUT5-insensitive Kv channels of known structure, including Kv1.2, the Kv1.2-Kv2.1 Chimera, and Kv4.2 [^3-7^](#_ENREF_3). AUT5 binding sites near the turret were not detected for the AUT5-insensitive channels (Fig. S12). This result provides strong computational support to the experimental observations that demonstrate the highly selective modulation of Kv3.1 and Kv3.2 by AUT5 is dictated by the structural features conferred by their specific amino-acid sequences near the turret.

**The S1-S2 may act as a hub to modulate the interaction between the turret and the S3-S4 loop.**

Although the turret sequence dictates gain and loss of AUT5 sensitivity among Kv3 variants, it is not clear how the turret is involved in the compounds’ MoA since it does not directly contribute to significant contacts of the compounds in the binding site (Fig. 2). To elucidate this problem, we performed a series of molecular dynamics (MD) simulations of the apo structure of Kv3.1 aimed at determining how mutation-induced structural perturbations of the turret could alter its interactions with neighboring regions and potentially neutralize AUT5 sensitivity (Fig. S20). Accordingly, the Kv3.1a wild type channel and the respective chimeras, 3.4x3.1Turret and S4R/A5G/S6N, were embedded in a fully hydrated phospholipid bilayer at 150 mM KCl solution, with K^+^ ions retained in the selectivity filter (SF) at their cryo-EM resolved three-dimensional positions (Fig. S20a). The 3.4x3.1Turret chimera involved the amino-acid replacements Q372R, N374S, S377R, A378G, S379N, E380D and H383D along the turret region of Kv3.1 and the S4R/A5G/S6N mutant involved only three of these amino-acid mutations S377R, A378G and S379N (Fig. 6).

Following initial minimization and thermalization, each macromolecular system was simulated for 500 ns, at constant temperature and pressure, neutral pH, and with no applied transmembrane electrostatic potential (Supplementary Methods). Steady-state analysis of the wild-type channel showed that structural fluctuations of the S3-S4 loop and the turret are dictated by direct amino acid contacts with the S1-S2 loop (Fig. S20b-c). Consequently, the S1-S2 loop appears to be part of an allosteric hub between the voltage-sensor and pore domain that is likely to impact the compound’s MoA. Indeed, MD simulations of the chimeras 3.1x3.4/Turret and S4R/A5G/S6N revealed that fluctuations of the S1-S2 loop are increased by mutations of the turret, which causes a significant reduction in the amino-acid contacts (Fig. S20c). The cumulative probabilities of amino acid contacts of the S1-S2 loop with the turret and S3-S4 loop $\sum p\left( n \right)$ are: 11.11 (WT), 9.40 (3.1x3.4/Turret) and 9.32 (S4R/A5G/S6N). These changes disrupt the allosteric coupling between the turret and S3-S4 loop (Fig. S20d). The separation distance between the turret and the S3-S4 loop increases significantly upon mutation (Fig. S20e). Particularly, the disruption of the interactions between the turret and the S3-S4 loop is larger for 3.4x3.1/Turret than S4R/A5G/S6N, which agrees with the observed effects of the mutations on the AUT5-induced positive modulation (Figs. 7 and 8).

Although our MD simulation results of the apo structure cannot be directly compared to the bound conformation of the channel, the revealed structural perturbations appear to be relevant for the MoA of the compounds. The mutation-induced structural rearrangements impact the physical coupling between turret and the S3-S4 loop but not the binding site cavity itself. Docking of AUT5 against all three simulated constructs of the apo Kv3.1 channel find solutions that largely resemble the bound configuration of the compound reported on Fig. 3. Docking solutions that reproduce the cryo-EM bound configuration of AUT5 are shown in Fig. S20f, with best structural superposition and interaction energies given by wild type (RMSD = 1.75 Å, E = -7.80 kcal/mol), 3.1x3.4Turret (RMSD = 1.31 Å, E = -8.30 kcal/mol), and S4R/A5G/S6N (RMSD = 2.30 Å, E = -7.10 kcal/mol). Altogether, the results corroborate the conclusion that the cap formed by close interactions of the turret and the S3-S4 loop on top of the binding site is fundamental for MoA (Movie #1). Therefore, disruption of turret and S3-S4 loop interactions via increased fluctuations of the S1-S2 loop emerges as a potential mechanism for the neutralizing effect of turret mutations on the compounds’ MoA.

**Supplementary Methods**

**Molecular Dynamics Simulations.**

Cryo-EM structure of the transmembrane domain of the Kv3.1a channel was obtained from the Protein Data Bank (ID: 7PHH). Missing residues in the transmembrane domain were inserted with aid of Modeller [^8^](#_ENREF_8). Modeller was used to generate the 3.1x3.4Turret chimera and its triple-mutant construction S4R/A5G/S6N. MD simulations were performed with the program NAMD 2.9. Langevin dynamics and Langevin piston methods were applied to keep constant the simulation temperature (300 K) and pressure (1 atm). The equations of motion were integrated using a multiple time-step algorithm [^9^](#_ENREF_9)^,^[^10^](#_ENREF_10). Short- and long-range forces were calculated every 1 and 2 time-steps respectively, with a time step of 2.0 *fs*. Chemical bonds between hydrogen and heavy atoms were constrained to their equilibrium value. Electrostatics were evaluated using Particle Mesh Ewald (PME) method with a grid spacing of 1.2 Å [^10^](#_ENREF_10). Periodic Boundary Conditions (PBC) were applied. The CHARMM36 force field was applied, and water molecules were described by the TIP3P model [^11^](#_ENREF_11)^,^[^12^](#_ENREF_12). Visual Molecular Dynamics (VMD) was used for simulation setup and analysis [^12^](#_ENREF_12)^,^[^13^](#_ENREF_13).

**Patch-clamp Electrophysiology of HEK-293 Cells Stably Expressing Kv3.4 Channels.**

The inducible HEK-293 cell line stably expressing the Kv3.4a channel was created commercially by SB Drug Discovery (Glasgow, UK) and kindly provided by Dr. N. Pilati (Autifony Srl, Italy) to conduct the experiments reported on Fig. S4. The cells were cultured using a standard protocol in tetracycline-free DMEM media with 10% FBS, 2mM L-glutamine, 50 units/ml penicillin, 50 µg/ml streptomycin, 2 mg/ml geneticin, and 4 µg/ml blasticidin. The cells were seeded onto glass coverslips 1-3 days before conducting patch-clamp recordings. Tetracycline (final concentration 0.01µg/ml) was added 18 - 24 hours prior to recording to induce the expression of the Kv3.4 channel. Concentration of tetracycline and incubation time was optimized to achieve the desired level of expression. Patch pipettes were pulled from borosilicate capillary glass (Warner Instruments) with a P-97 micropipette puller (Sutter Instrument). The resistance of patch pipettes was 2 – 3 MΩ after fire polishing. Whole-cell patch-clamp recording was performed using an Axopatch 200B amplifier (Molecular Devices) and Digidata 1440A analogue-to-digital converter (Molecular Devices). Signals were low-pass filtered at 2 kHz and sampled at 5x this frequency. Series resistance was compensated 80-90%, and passive leak current and capacitive transients were subtracted online with a P/4 protocol. The pCLAMP 10 suite (Molecular Devices) was used for acquisition and analysis. The extracellular bath solution contained (in mM) 137 NaCl, 4 KCl, 1.8 CaCl_2_, 1 MgCl_2_, 10 HEPES, and 10 D-glucose, pH 7.3, adjusted with NaOH; and the intracellular pipette solution contained (in mM) 120 KCl, 31.3 KOH, 5.37 CaCl_2_, 1.75 MgCl_2_, 10 EGTA, 10 HEPES, and 4ATPMg, pH 7.2, adjusted with KOH.

**Movie #1.**

Morphing animation of the apo and AUT5-bound conformations of Kv3.1a. AUT5 is shown in red occupying the interfacial binding pocket between two neighboring subunits. From the voltage sensing domain, the S1 and S2 helices are shown in blue and the S3 and S4 helices are shown in purple. From the pore domain, the S5 and S6 helices are shown in cyan. Upon AUT5 binding the extracellular S5-S6 linker (Turret) folds toward the extracellular S1-S2 linker of the voltage sensing domain to trap the compound in its pocket.

**Supplementary** **References**

1 Chi, G. *et al.* Cryo-EM structure of the human Kv3.1 channel reveals gating control by the cytoplasmic T1 domain. *Nat Commun* **13**, 4087 (2022). <https://doi.org:10.1038/s41467-022-29594-w>

2 Mirdita, M., Schutze, K., Moriwaki, Y., Heo, L., Ovchinnikov, S. & Steinegger, M. ColabFold: making protein folding accessible to all. *Nat Methods* **19**, 679-682 (2022). <https://doi.org:10.1038/s41592-022-01488-1>

3 Long, S. B., Tao, X., Campbell, E. B. & MacKinnon, R. Atomic structure of a voltage-dependent K+ channel in a lipid membrane-like environment. *Nature* **450**, 376-382 (2007).

4 Long, S. B., Campbell, E. B. & MacKinnon, R. Crystal Structure of a Mammalian Voltage-Dependent Shaker Family K+ Channel. *Science* **309**, 897-902 (2005).

5 Kise, Y. *et al.* Structural basis of gating modulation of Kv4 channel complexes. *Nature* **599**, 158-164 (2021). <https://doi.org:10.1038/s41586-021-03935-z>

6 Matthies, D. *et al.* Single-particle cryo-EM structure of a voltage-activated potassium channel in lipid nanodiscs. *Elife* **7** (2018). <https://doi.org:10.7554/eLife.37558>

7 Reddi, R., Matulef, K., Riederer, E. A., Whorton, M. R. & Valiyaveetil, F. I. Structural basis for C-type inactivation in a Shaker family voltage-gated K(+) channel. *Sci Adv* **8**, eabm8804 (2022). <https://doi.org:10.1126/sciadv.abm8804>

8 Webb, B. & Sali, A. Protein Structure Modeling with MODELLER. *Methods Mol Biol* **2199**, 239-255 (2021). <https://doi.org:10.1007/978-1-0716-0892-0_14>

9 Phillips, J. C. *et al.* Scalable molecular dynamics with NAMD. *J Comput Chem* **26**, 1781-1802 (2005).

10 Darden, T., York, D. & Pedersen, L. Particle mesh Ewald - An Nlog(N) method for Ewald sums in large systems. *J Chem Phys* **98**, 10089-10092 (1993).

11 Huang, J. & MacKerell, A. D., Jr. CHARMM36 all-atom additive protein force field: validation based on comparison to NMR data. *J Comput Chem* **34**, 2135-2145 (2013). <https://doi.org:10.1002/jcc.23354>

12 Jorgensen, W. L., Chandrasekhar, J., Madura, J. D., Impey, R. W. & Klein, M. L. Comparison of simple potential functions for simulating liquid water. *J Chem Phys* **79**, 926-935 (1983).

13 Humphrey, W., Dalke, A. & Schulten, K. VMD: visual molecular dynamics. *J Mol Graph* **14**, 33-38, 27-38 (1996).
