# Supplementary material for: The binding and mechanism of a positive allosteric modulator of Kv3 channels
Source: Nat Commun. 2024 Mar 21;15:2533. doi: 10.1038/s41467-024-46813-8 (PMC10957983; doi:10.1038/s41467-024-46813-8)
Supplement: Supplementary file 3 — Description of Additional Supplementary Files [file 41467_2024_46813_MOESM3_ESM.pdf]

File Name: Supplementary Movie 1

Description: Morphing animation of the apo and AUT5-bound conformations of Kv3.1a. AUT5 is shown in red occupying the interfacial binding pocket between two neighboring subunits. From the voltage sensing domain, the S1 and S2 helices are shown in blue and the S3 and S4 helices are shown in purple. From the pore domain, the S5 and S6 helices are shown in cyan. Upon AUT5 binding the extracellular S5-S6 linker (Turret) folds toward the extracellular S1-S2 linker of the voltage sensing domain to trap the compound in its pocket.
