# Supplementary material for: The binding and mechanism of a positive allosteric modulator of Kv3 channels
Source: Nat Commun. 2024 Mar 21;15:2533. doi: 10.1038/s41467-024-46813-8 (PMC10957983; doi:10.1038/s41467-024-46813-8)
Supplement: Supplementary file 5 — Reporting Summary [file 41467_2024_46813_MOESM5_ESM.pdf]

Corresponding author(s): Manuel Covarrubias
Last updated by author(s): 02/19/2024

Reporting Summary

Nature Portfolio wishes to improve the reproducibility of the work that we publish. This form provides structure for consistency and transparency in reporting. For further information on Nature Portfolio policies, see our Editorial Policies and the Editorial Policy Checklist.

Statistics

For all statistical analyses, confirm that the following items are present in the figure legend, table legend, main text, or Methods section.

n/a	Confirmed	
☐	☒	The exact sample size (*n*) for each experimental group/condition, given as a discrete number and unit of measurement
☐	☒	A statement on whether measurements were taken from distinct samples or whether the same sample was measured repeatedly
☐	☒	The statistical test(s) used AND whether they are one- or two-sided<br>*Only common tests should be described solely by name; describe more complex techniques in the Methods section.*
☒	☐	A description of all covariates tested
☐	☒	A description of any assumptions or corrections, such as tests of normality and adjustment for multiple comparisons
☐	☒	A full description of the statistical parameters including central tendency (e.g. means) or other basic estimates (e.g. regression coefficient) AND variation (e.g. standard deviation) or associated estimates of uncertainty (e.g. confidence intervals)
☐	☒	For null hypothesis testing, the test statistic (e.g. *F*, *t*, *r*) with confidence intervals, effect sizes, degrees of freedom and *P* value noted<br>*Give P values as exact values whenever suitable.*
☒	☐	For Bayesian analysis, information on the choice of priors and Markov chain Monte Carlo settings
☒	☐	For hierarchical and complex designs, identification of the appropriate level for tests and full reporting of outcomes
☒	☐	Estimates of effect sizes (e.g. Cohen's *d*, Pearson's *r*), indicating how they were calculated

Our web collection on statistics for biologists contains articles on many of the points above.

Software and code

Policy information about availability of computer code

Data collection	For electrophysiology, Clampex (pClamp 10.7). For cryo-EM data collection, EPU version 2 was used to collect the electron micrographs. Molecular dynamics simulations were performed using NAMD version 2.12 . Molecule conversion occurred with OpenBabel 3.0.0 . Molecular docking calculations were performed by AutoDockVina 1.1.2 . Docking and simulation programs are open access for research purposes.
Data analysis	For electrophysiology (data analysis and statistics), Clampfit (pClamp 10.7), OriginPro 10.0. Cryosparc 2.14 was used to process the datasets, and the EM reconstructions were analysed for model building with Wincoot and Phenix, as stated in the manuscript methods. Data analysis was performed using Visual Molecular Dynamics (VMD) version 1.9.2 and Python (version 3.5) scripts. Docking and simulation programs open access for research purposes.

For manuscripts utilizing custom algorithms or software that are central to the research but not yet described in published literature, software must be made available to editors and reviewers. We strongly encourage code deposition in a community repository (e.g. GitHub). See the Nature Portfolio guidelines for submitting code & software for further information.

Data

Policy information about availability of data

All manuscripts must include a data availability statement. This statement should provide the following information, where applicable:

- Accession codes, unique identifiers, or web links for publicly available datasets
- A description of any restrictions on data availability
- For clinical datasets or third party data, please ensure that the statement adheres to our policy

The cryo-EM maps of the human Kv3.1a structures (bound conformations) and the corresponding atomic coordinates have been deposited in the Electron Microscopy Data Bank and the Protein Data Bank under the accession codes EMD-13417 / 7PHI (APO), EMD-18659 / 8QUC (AUT1 bound) and EMD-18660 / 8QUD (AUT5 bound). These materials will be publicly available upon release, which will occur upon the acceptance and publication of this manuscript. Source data for electrophysiological experiments are provided with this paper (Supplemental Figs. S1-S5, Figs. S13-S19, and Table S4). Source data and validation for cryo-EM experiments are provided with this paper (Supplemental Fig. S6; Table S1). Source data and validation for molecular dynamics simulations are provided with this paper (Supplemental Fig. S20). Source data and validation for docking calculations are provided with this paper (Fig. 3, Supplemental Figs. S11-S12). The paper includes a detailed Data Availability Statement.

Research involving human participants, their data, or biological material

Policy information about studies with human participants or human data. See also policy information about sex, gender (identity/presentation), and sexual orientation and race, ethnicity and racism.

Reporting on sex and gender	N/A
Reporting on race, ethnicity, or other socially relevant groupings	N/A
Population characteristics	N/A
Recruitment	N/A
Ethics oversight	N/A

Note that full information on the approval of the study protocol must also be provided in the manuscript.

Field-specific reporting

Please select the one below that is the best fit for your research. If you are not sure, read the appropriate sections before making your selection.

☒ Life sciences ☐ Behavioural & social sciences ☐ Ecological, evolutionary & environmental sciences

For a reference copy of the document with all sections, see nature.com/documents/nr-reporting-summary-flat.pdf

Life sciences study design

All studies must disclose on these points even when the disclosure is negative.

Sample size	The sample sizes of electrophysiological experiments were determined from the our multi-year experience conducting similar studies that have characterized the impact of mutations on the biophysical properties of ion channels, and the potential impact of variations between batches of oocytes (see Replication below). The following citations of the authors' work are examples that have helped determined the necessary sample size: Liang et al., PloS One, 10(11):e0143363 doi: 10.1371/journal.pone.0143363, 2015; Chi et al., Nat Commun., 13(1):4087. doi: 10.1038/s41467-022-29594-w, 2022; Clatot et al., PNAS, 121(3):e2307776121. doi: 10.1073/pnas.2307776121 2024.
Data exclusions	Generally, no data were excluded, and all data points were plotted. Electrophysiological recordings were curated before data analysis. A recording may not be included in the analysis if there was a technical issue, such as an excessive leak current, poorly voltage clamped currents or a significant voltage offset. For single particle cryo-EM analyses, particles were excluded which did not improve the map quality. This utilises standard classification procedures in CryoSPARC and is standard practice for structure determination by cryo-EM.
Replication	To ensure replication and minimize a covariate effect, most electrophysiological experiments were conducted with at least two independent batches of Xenopus oocytes (from two separate frogs). The properties of the wild-type controls and some mutants were replicated over a period of more than ten years with several dozen batches of oocytes. For cryo-EM, purification quality controls such as size exclusion chromatography or SDS PAGE were at least repeated twice for each condition and at least 4 cryo-EM grids were prepared under identical conditions. Structure determination was completed once for each condition. Two ligand-bound cryo-EM structures presented in this study are near-replicates of each other as the ligands are structurally very similar.
Randomization	Electrophysiological data were not randomized. A possible covariate due to variations between batches of oocytes was accounted for as explained above. For cryo-EM analyses, randomization is achieved by assigning particles randomly to half-maps for determining the resolution by fourier shell correlation. Molecular dynamics simulations start with a random distribution of atom velocities.

Blinding	No blinding was applied. During a recording session for any given mutant in the absence and presence of compound, we systematically included recordings of the wild type control in the absence and presence of compound. Compounds were tested independently on separate recording sessions that included several independent batches of oocytes. A study design that included several orthogonal experiments and approaches (as shown in the manuscript) yielded replicable results over a period of several years.

Reporting for specific materials, systems and methods

We require information from authors about some types of materials, experimental systems and methods used in many studies. Here, indicate whether each material, system or method listed is relevant to your study. If you are not sure if a list item applies to your research, read the appropriate section before selecting a response.

Materials & experimental systems

n/a	Involved in the study
☒	☐ Antibodies
☐	☒ Eukaryotic cell lines
☒	☐ Palaeontology and archaeology
☐	☒ Animals and other organisms
☒	☐ Clinical data
☒	☐ Dual use research of concern
☒	☐ Plants

Methods

n/a	Involved in the study
☒	☐ ChIP-seq
☒	☐ Flow cytometry
☒	☐ MRI-based neuroimaging

Eukaryotic cell lines

Policy information about cell lines and Sex and Gender in Research

Cell line source(s)	HEK293 cell line stably expressing human Kv3.4. This cell line was created commercially by SB Drug Discovery (Glasgow, UK). HEK293F "Expi" cells (Expi293F, Thermo Fisher, A14527) for overexpression of human Kv3.1a using BacMam system as stated under Methods.
Authentication	Electrophysiological authentication to demonstrate replicable expression of Kv3.4 currents with their expected biophysical profile (Supplemental Fig. S4). The HEK293F "Expi" cells were purchased as an authenticated cell line from Thermo Fisher.
Mycoplasma contamination	Cells were not tested for mycoplasm. There was, however, no evidence of contamination.
Commonly misidentified lines (See ICLAC register)	No commonly misidentified cell lines were used in this study

Animals and other research organisms

Policy information about studies involving animals; ARRIVE guidelines recommended for reporting animal research, and Sex and Gender in Research

Laboratory animals	Xenopus laevis. Animals themselves were not used. As described under Methods, oocytes were harvested from adult female frogs and used as a heterologous expression system. The exact age of the frogs we get from the vendor (Xenopus1.com) is unknown. However, they are adults with ovaries capable of producing healthy oocytes.
Wild animals	No wild animals were used in this study
Reporting on sex	As indicated above, female frogs were used to harvest oocytes. The frogs themselves are not used in this study.
Field-collected samples	No field-collected samples were used
Ethics oversight	Institutional Animal Care and Use Committee (IACUC) of Thomas Jefferson University

Note that full information on the approval of the study protocol must also be provided in the manuscript.

Plants

Seed stocks	N/A
Novel plant genotypes	N/A
Authentication	N/A
